# Weak Hearts

**Published:** 1889-11

**Authors:** 


### WEAK HEARTS.

A weak heart seems to be decidedly more practically inconvenient than a weak head. If a man or a woman be a little feeble about the region of the brain it is generally of little moment. Some post or other will be provided if the conduct be respectable; and lack of brains is too commom to excite any particular attention either in the person concerned or in those about him. But a weak heart insists upon putting itself in evidence at all sorts of convenient and inconvenient times. If its possessor finds himself rather late for his morning train, and makes a "spurt" to recover lost time, the exertion is usually followed by such a "bad quarter of an hour" that he resolves in future rather to lose a dozen trains than to risk temporary suffocation or permanent syncopo again. The practical evils which are associated with a feeble heart are innumerable, and will readily suggest themselves to those who possess so unsatisfactory a pumping engine. Weak hearts are by no means so common as is often supposed. Many a man whe thinks he has got one is merely dyspeptic ; many a woman owes her symptoms to tight lacing or insufficient feeding. If the dyspepsia be cured, or the tight lacing be dispensed with, the symptoms of heart weakness will disappear. Even when the heart is genuinely "weak," the weakness is not always due to special disease of that organ. It may be only part of a general weakness of the whole system, which is easily curable.

---

Statistics show that whiskey causes, on an average, 1,300 funerals per day in the United States. There are, also, 3,000 penitentiary convicts and 285,000 occasional prisoners developed each year by the influence of liquor.

---

The Southern Pacific Railway Company has closed all bars where intoxicating liquors are sold on its lines, and has established, for its 400 employees, a system of hot, temperance lunches, at the low cost of sixteen cents per day.

---

There are not two standards of right and wrong—one for men and one for women. Nor are there two standards of morality. It is as wrong for a man to be intemperate and unchaste as for a woman, no matter what a depraved public sentiment may declare to the contrary. And this we must teach our children, that there is but one law of right for both man and woman, which is supreme, and from which there is no appeal.

